# Craniospinal Surgery in Hajdu-Cheney Syndrome: A Review of Case Reports

**DOI:** 10.7759/cureus.20501

**Published:** 2021-12-18

**Authors:** Cody J Falls, Paul S Page, James A Stadler

**Affiliations:** 1 Neurological Surgery, University of Wisconsin School of Medicine and Public Health, Madison, USA; 2 Neurological Surgery, University of Wisconsin, Madison, USA

**Keywords:** orthopedic spine surgery, surgery spine, pediatric spine, bone disorder, spine deformity surgery

## Abstract

Hajdu-Cheney syndrome (HCS) is a rare metabolic bone disorder that results in severe osteoporosis and various skeletal deformities. Craniospinal pathology is commonly associated with it, but surgical management is challenging due to the distorted anatomy, reduced bone strength, and fusion failure due to osteolysis. Hence, the surgical difficulty in these patients requires careful consideration. In this study, we systematically review all published operative cases and complications to provide a comprehensive review pertaining to the spine and/or cranium in patients with HCS. By highlighting these cases and their associated complications, we aim to prepare practitioners who treat this difficult pathology.

## Introduction and background

According to the National Organization of Rare Disorders (NORD), roughly 80 cases of Hajdu-Cheney syndrome (HCS) have been reported in the medical literature. The condition is known to be strongly associated with a gain-of-function mutation in the NOTCH2 transmembrane protein, though the exact mechanism of disease manifestations is still under investigation [1].

The most characteristic finding of HCS is acro-osteolysis, though various skeletal manifestations are commonly seen, including dysmorphic facial features, joint laxity, and cranial deformities. Neurological, cardiac, and renal abnormalities may also be observed. Most significantly, patients often have severe osteoporosis. This predisposes them to fractures and early-onset progressive spinal deformity leading to local impingement and spinal concerns including radiculopathy, myelopathy, scoliosis, basilar invagination, and brainstem compression. While treatment with bisphosphonate therapy and bisphosphonates combined with teriparatide has been reported, it is unclear if these therapies bring any benefits to patients with HCS, and progressive structural concerns have warranted spinal surgery in many of the affected patients [1,2].

The significant osteoligamentous abnormalities in HCS are concerning for surgeons caring for these patients. Many post-surgical complications may arise if craniospinal operations are carried out in standard fashion, without consideration of the unique challenges in patients with HCS. In this article, we aim to analyze all known operative cases of the spine and/or cranium in patients with HCS, the techniques used in these operations, and their corresponding outcomes and complications.

## Review

The major scholarly databases PubMed, Google Scholar, Web of Science, and SCOPUS were searched for all operative cases pertaining to Hajdu-Cheney Syndrome. The keywords “Hajdu-Cheney Syndrome” and “HCS” were paired with all variations of the terms “operation” and “surgery”. All articles and abstracts were reviewed and any cases involving operative management pertaining to the cranium or spine were included. Articles originally published in a language other than English were also included if there was an English-translated version available. This study was deemed exempt by our facility's Institutional Review Board. A summary of relevant cases in chronological order is provided in Table 1.

Case presentations (chronological order)

**Table 1:** Known cranial and spinal operative cases in HCS patients with case and operative details *Age at the time of surgery; **Details were not provided; ***If left blank, information not included in the article; ****Addition of BMP and bone stimulator postoperatively ACDF: anterior cervical discectomy and fusion; BMP: bone morphogenetic protein; HCS: Hajdu-Cheney syndrome

Article (authors and year)	Age*, Sex	Presenting issue	Cranial/spinal pathology	Operation	Complication(s)	Instrumentation intact?	Postop orthosis	Follow-up
Chodoroff et al., 1984 [3]	30, F	Cervical pain, lower extremity weakness, difficulty ambulating	Posterior fossa compression	Foramen magnum decompression, C1 laminectomy, posterior cervical fusion**	None		Halo vest	2 months
Herscovici et al., 1990 [4]	10, M	Incidental finding	Spondylolisthesis of C2 on C3	C1-C3 posterior fusion	Resorption of bone graft	Yes	Halo vest	1 year
Herscovici et al., 1990 (reoperation) [4]	18, M	Radiculopathy	C3-C4 instability	Occiput-C6 posterior fusion	Osteolysis of the fusion mass	Yes		Unclear
Tanimoto et al., 1996 [5]	41, F	Quadriparesis and sensory deficits (post-trauma)	Basilar invagination, atlantoaxial dislocation, Chiari 1 malformation, syringomyelia of C2 and C5-T6 regions	Suboccipital craniectomy and C1 laminectomy with duraplasty-OC fusion	None	Yes	Modified Philadelphia collar	3 months
Faure et al., 2002 [6]	7, M	Headaches, cervical pain, autonomic sx, paravertebral contracture	C5-T2 syringomyelia, basilar impression	Occipital craniectomy, C1 laminectomy, and duraplasty	Immediate: phonation disorder, loud voice; 5 months: worsening platybasia; 9 months: worsening platybasia and syrinx enlargement	N/A	None	2.5 years
Binder et al., 2006 [7]	21, M	Cervical pain, paresthesia, and spasm	Basilar invagination and basilar compression	“Surgical stabilization of C-spine”**	Wound infection, osteomyelitis, cord edema, and formation of 2 syringes			
Murtagh-Schaffer and Moquin, 2008 [8]	42, M	Paresthesia of upper extremities (post-trauma)	Fractures of C5-C6 and C7-T1; perched facets at C6-C7	C5-T1 ACDF, C5-T4 posterior fusion	Fusion failure and loss of correction	Yes	“Cervical orthosis”	
Murtagh-Schaffer and Moquin, 2008 (reoperation) [8]	42, M	Found during hospitalization for an unrelated issue	Fusion failure and loss of deformity correction	Extension of posterior fusion (C2-T4)****	None	Yes	“Cervical orthosis”	6 months
Mattei et al., 2015 [9]	65, F	Worsening gait, lower extremity weakness, paresthesia of upper and lower extremities	Cervical stenosis, spondylolisthesis of C6 on C7, fracture of multiple cervical lamina and pedicles, cervical kyphosis (50 degrees)	C6 corpectomy, multilevel cervical diskectomy, and wide-opening of the posterior longitudinal ligament. C2-T1 ACDF, C2-T3 posterior fusion	None	Yes	Halo vest	1 year
Woon and Mardjetko, 2018. [10]	20, F	Loss of hearing and cervical pain with coughing	Basilar invagination, hydrocephalus, multiple compression fractures, mid-cervical kyphosis, thoracic lordoscoliosis	Suboccipital craniectomy, C1-C4 laminectomy, occiput-T2 fixation	Suture diastasis with frontoparietal sag	Yes	Halo vest	2 months
Woon and Mardjetko, 2018 (reoperation) [10]	20, F	Decreased hearing, posterior headaches, gargled speech, jaw pain, regurgitation, changes in facial features	Suture diastasis with frontoparietal sag. Anterior-caudad translation of parietal bones	Extension of posterior OC fusion construct to parietal bones via titanium rods, titanium mesh fixation of frontal and parietal bones	None	Yes		18 months
Vissarionov et al., 2019 [11]	7, F	Back pain post-trauma	T5 vertebral body burst fracture. T5 spinal stenosis. T3, T8, L3 vertebrae compression fractures. Thoracic kyphosis	Post-traumatic deformity correction, spinal stenosis elimination, and stabilization of spine via T4, T6, and T7 transpedicular fixation with posterior instrumentation and bone autograft placement	No complications for 3 years. At 4 years, worsening thoracic kyphosis noticed; an abrupt decrease in height of the anterior column of the T8 vertebral body. Distal junctional kyphosis below inserted instrumentation	Yes	Rigid hyperextension thoracolumbar brace (1.5 years, 18 hours/day)	4 years
Vissarionov et al., 2019 [11]	11, F	Thoracic kyphosis	An abrupt decrease in height of the anterior column of the T8 vertebral body. Distal junctional kyphosis below inserted instrumentation	Deformity correction with posterior construct extension to T9 (T4-T9) via transpedicular screws and rods with bone autograft placement	None	Yes	Thoracolumbar brace	8 months
Falls et al., 2021 [12]	38, F	Cervical/upper thoracic back pain. Worsening thoracic scoliosis	Thoracic scoliosis (60 degrees), thoracic hypokyphosis, compensatory cervical kyphosis	Posterior facetectomies at C4/5, C5/6, and C6/7 with anterior C5 and C6 corpectomy and C4 to C7 anterior fusion with expandable cage placement. C4-C7 posterior fusion. C4-T11 deformity correction, bilateral facetectomies from T5-T8, and C4-T11 posterior fusion	None	Yes	None	1 year
Falls et al., 2021 [12]	25, F	Headaches, stridulous breathing, suboccipital neck pain, unsteadiness, facial numbness/tinging, blurry vision, and subjective hearing loss	Basilar invagination with brainstem compression; upper cervical syrinx	Occiput to C4 posterior fusion with the extension of construct superiorly to parietal bones. Arthrodesis of cranial sutures with cranial plating	None	Yes	Craniocervical bracing	1 year

Case 1 [3]

Background: Chodoroff et al. published an article in 1984 describing a 30-year-old female with HCS who presented with cervical pain, lower extremity weakness, and difficulty ambulating. The evaluation revealed posterior fossa compression requiring operative management given progressive neurologic decline.

Operation: foramen magnum decompression with C1 laminectomy and posterior cervical fusion. Operative details were not provided in the article. The patient was placed in a halo vest postoperatively.

Outcome/complication(s): two months postoperatively, the patient continued to have difficulties with ambulation and self-care. The article details the rehabilitation process but very little information is provided otherwise regarding her management and postoperative course.

Case 2 [4]

Background: Hersovici et al. published a paper in 1990 describing the findings in a young girl with HCS who, at seven years of age, was incidentally found to have a “spondylolytic type of defect” at the pedicles of C2 with anterior displacement of C2 on C3. The patient reported no associated neurologic symptoms, initially. During subsequent clinic follow-ups, her cervical instability was determined to be progressing rapidly on flexion and extension X-rays. Hence, a posterior cervical fusion was recommended. 

Operation #1: C1-C3 posterior fusion with iliac bone graft. The patient was placed in a halo vest postoperatively.

Outcome/complication(s): at approximately one year postoperatively (11 years, five months of age), follow-up X-rays demonstrated resorption of the bone graft at C1-C2. She was readmitted for augmentation of fusion mass (operative details were not included).

At 18 years old, the patient was seen for cervical pain. The evaluation revealed chronic cervical radiculopathy. Flexion and extension radiographs demonstrated new cervical instability at C3-C4 representing adjacent segment disease, and surgical stabilization was recommended.

Operation #2: occipitocervical fixation from occiput to C6 using iliac crest with donor bone graft.

Outcome/complication(s): follow-up X-rays revealed osteolysis of fusion mass. The authors noted that the patient was asymptomatic “at present” but remained unclear on time frames. No further follow-up was provided.

Case 3 [5]

Background: Tanimoto et al. published a case report in 1996 detailing the case of a 41-year-old female with HCS who presented with mild quadriparesis and sensory deficits after a motor vehicle accident. A neurologic exam revealed bilateral hyperactive deep tendon reflexes and hypesthesia in bilateral upper extremities. Imaging showed basilar invagination, atlantoaxial dislocation with superior displacement of the dens, a Chiari 1 malformation, and syringomyelia at C2 and from C5-T6.

Operation: foramen magnum decompression with C1 laminectomy and duraplasty followed by occipitocervical fixation (levels were not included in the paper) with iliac bone graft and titanium wires. A modified Philadelphia collar was used postoperatively.

Outcome/complication(s): three months postoperatively, there was an improvement of the quadriparesis and sensory deficits. The patient reported being able to ambulate much better. Imaging revealed a well-decompressed foramen magnum, complete resolution of the C2 syrinx, and reduction of the C5-T6 syrinx.

Case 4 [6]

Background: Faure et al. published a case report in 2002 describing a seven-year-old male with known HCS that presented with occipital headaches, irregularly occurring autonomic symptoms (sweating/paleness), cervical pain, and paravertebral contractures. Motor and sensory evoked potentials were normal. Imaging revealed significant syringomyelia with surrounding edema at C5-T2. Basilar impression was also apparent and progressing rapidly based on prior imaging. Due to the rapidly progressing course and spinal cord edema with syrinx formation in close proximation to symptom onset, operative management was recommended.

Operation: occipital craniectomy, C1 laminectomy, and duraplasty. No occipitocervical fusion was performed. Postoperative external immobilization was not used.

Outcome/complication(s): shortly after the operation, his headache and neck stiffness were improved, though a phonation disorder and loud voice developed. An evaluation revealed no explanation. Five months postoperatively, the patient appeared well. Imaging revealed syrinx regression but worsening platybasia (basal angle 154 degrees). At nine months postoperatively, the patient’s headache and cervical pain returned. Imaging revealed further worsening of platybasia and syrinx recurrence. Development of thoracolumbar scoliosis was also apparent. The patient was placed in a Milwaukee corset and bisphosphonate infusions were started. He was managed in an external orthosis for over two years. His headaches, cervical pain, and phonation disorder improved and his neurologic exam became normal. Imaging revealed stabilization of platybasia and enlargement of the syrinx cavity. The patient was managed with a continuation of orthosis and syrinx monitoring.

Case 5 [7]

Background: in this abstract presented by Binder et al. in 2006, the authors describe a 21-year-old male with a known history of HCS who presented with pain, paresthesia, and cervical muscle spasms secondary to progressive basilar invagination and brainstem compression.

Operation: “Surgical stabilization of the C-spine”.

Outcome/complication(s): postoperatively, the patient developed a wound infection that progressed to osteomyelitis, spinal cord edema, and formation of two syrinxes. Instrumentation removal was declined. His myelopathy worsened as demonstrated by increased spasticity; this was managed medically, with no mention of reoperation.

Case 6 [8]

Background: Murtagh-Schaffer and Moquin published a paper in 2008 describing a 42-year-old man with HCS who presented to the emergency department after a motor vehicle collision. His only neurological deficit was paresthesia of the upper extremities. He was subsequently found to have fractures at C5-C6 and C7-T1 and perched facets at C6-C7.

Operation #1: C5-T1 anterior diskectomy and fusion with patella allograft; C5-T4 posterior fusion with crushed cancellous allograft and local autograft. He was discharged with a cervical orthotic.

Outcome/complication(s): The patient was admitted at a later date (time frame unclear) for an unrelated issue. Fusion failure and loss of correction were noted on imaging at this time.

Operation #2: a posterior-only revision with the extension of the fusion to C2 (now C2-T4 fusion). Bone morphogenetic protein (BMP) was used with allo/autograft. The patient was again discharged with a cervical orthotic. A bone stimulator was used postoperatively.

Outcome/complication(s): at six months postoperatively, full fusion was maintained and instrumentation remained in the proper positioning. At this time, the patient reported doing well without neck pain or any neurological symptoms.

Case 7 [9]

Background: Mattei et al. published an article in 2015 detailing the case of a 65-year-old female with HCS who presented with months of progressively worsening gait and lower extremity weakness as well as three days of paresthesia of the upper and lower extremities that had been brought on by a sudden movement of her neck. Neurological exam showed abnormal Hoffman and Babinski signs, and sustained clonus was apparent. MRI revealed cervical stenosis, most prominently at C6, and CT showed spondylolisthesis of C6 on C7 as well as fractures of multiple lamina and pedicles resulting in cervical kyphotic deformity of 50 degrees. Due to the degree of cervical kyphosis and myelopathy, surgery was recommended and carried out in two stages.

Operation: stage 1 - anterior C6 corpectomy and multi-level C2-T1 discectomy (C2-T1) with the wide opening of the posterior longitudinal ligament. This was followed by 24 hours of halo-traction to reduce the cervical kyphotic deformity.

Stage 2a - bone allograft placement at the C6 corpectomy site and anterior cervical interbody fusion of C2-T1 with bone allograft and plating. Stage 2b - posterior C2-T3 fusion with rigid instrumentation. A halo vest was utilized postoperatively.

Outcome/complication(s): at the one-year follow-up, significant neurological improvement was noted. The patient reported better hand coordination and strength. She also reported no neck pain. At this time, imaging showed all instrumentation was in correct positioning and fusion was progressing appropriately.

Case 8 [10]

Background: Woon and Mardjetko published an article in 2018 describing the findings in a 20-year-old female with HCS who presented with loss of hearing and cervical pain with coughing. Imaging revealed severe osteoporosis, multiple compression fractures, basilar invagination, hydrocephalus, mid-cervical kyphosis, and thoracic scoliosis with lordosis. Management with halo traction was attempted but stopped when the patient developed transient motor weakness.

Operation #1: suboccipital craniectomy, C1-C4 laminectomy, C3-T1 osteotomies, and occiput-T2 posterior fusion. The fusion was then carried out with a posterior plate, lateral mass screws, and pedicle screws augmented by structural allograft rib struts, demineralized bone matrix (DBM), and BMP. The halo vest was then reapplied.

Outcome/complication(s): postoperatively, the patient showed improvement in swallowing and hearing. She developed Bell’s palsy, which was treated with a course of corticosteroids. Two months after the surgery, the halo vest was removed and transitioned to the cervicothoracic orthosis. Following the vest removal, the patient complained of decreased hearing, posterior headaches, and right ear pain. An evaluation revealed no intracranial abnormalities, and all instrumentation was in correct positioning. Three days later, the patient became further symptomatic with increased speech effort and “wet”, garbled speech. Nausea, jaw pain, regurgitation, and changes in facial features were also noted. Imaging revealed suture diastasis that was increased in the upright position and decreased when supine. There was also a frontoparietal sag. This was determined to be due to anterior-caudad translation of the parietal bones away from the occipital bone, particularly prominent in upright positioning. The patient was subsequently placed back into her halo vest and taken for reoperation.

Operation #2: extension of the prior fusion to the frontal bones. Long troughs were created in the parietal bones with a burr. Titanium rods were then contoured to fit these troughs, affixed to connectors, and connected to the prior instrumentation construct. Titanium mesh was placed over the rods and anchored by titanium screws, connecting the frontal and parietal bones. This was augmented by strips of allograft rib and DBM.

Outcome/complication(s): at 18 months postoperatively, the patient continued to do well with the resolution of cranial nerve symptoms. Imaging showed instrumentation in correct positioning.

Case 9 [11]

Background: Vissarionov et al. published an article in 2019 detailing the case of a seven-year-old female with HCS who presented after a fall with back pain in her thoracic spine radiating to the lumbar spine. Her neurologic exam was unremarkable. Imaging revealed a burst fracture of the T5 vertebral body and compression fractures of T3, T8, and L3 vertebrae. Spinal stenosis was also present at T5. Densitometry was carried out revealing poor bone density. Due to the degree of spinal injury and potential for imminent neurologic instability, the patient was taken for an emergency operation.

Operation: T4-T7 posterior spinal fusion with autograft. Thoracic deformity correction was carried out via pedicular screws and rods bent to achieve physiologic kyphosis. The fractured levels (T3 and T8) were not included in this construct. The patient was started on regular bisphosphonate infusions and a rigid hyperextension thoracolumbar brace was used postoperatively.

Outcome/complication(s): Three years postoperatively, the patient continued to do well with no complaints. Imaging revealed an appropriate spinal alignment and all instrumentation remained in the correct positioning.

Four years postoperatively, worsening thoracic kyphoscoliosis was noticed. Imaging revealed an abrupt decrease in the anterior column height of the T8 vertebral body. Thoracic kyphosis was 54 degrees and the thoracic dextroscoliosis from T3-T10 was 25 degrees. Due to the patient’s age, scoliotic deformity, and progressive junctional kyphosis, spinal reconstruction was recommended.

Operation #2: extension of the posterior spinal fusion construct (now T4-T9) with bone autograft placement.

Outcome/complication(s): eight months postoperatively, the patient continued to do well. Imaging showed no loss of correction and all instrumentation was intact.

Case 10 [12]

Background: the authors of this paper published an article in 2021 detailing the case of a 25-year-old female with HCS who presented with several years of headache, stridulous breathing, and suboccipital neck pain. She previously had a ventriculoperitoneal shunt placed, which had initially reduced the intensity of her headaches but had not resolved them. The patient also reported decreasing balance, unsteadiness, facial numbness/tinging, blurry vision, and subjective hearing loss. Her physical exam was notable for limited cervical range of motion and signs of myelopathy manifested by left-sided hyperreflexia, abnormal Hoffman sign, and clonus. Imaging revealed progressive basilar invagination and associated brainstem compression. An upper cervical syrinx was also present that showed continued progression. Due to the significant nature of her craniocervical instability as well as the progressive worsening of her upper cervical syringomyelia, operative management was recommended. Prior to surgery, her shunt function was confirmed with normal intracranial pressure monitoring results.

Operation: craniocervical fusion extending from the frontal bones to C4 with posterior instrumentation, local autograft, nonstructural allograft, and BMP. Hinged rods from the occipital plate and cervical screws were extended superiorly and anchored to parietal bones via rod wires fixated to the parietal bones through burr holes, and multiple sheets of cranial mesh were used to extend the fusion to the frontal bones. The sagittal suture, bilateral lambdoid sutures, and bilateral coronal sutures were arthrodesed and fused with nonstructural allograft and BMP. She was placed in a modified halo vest for six months postoperatively.

Outcome/complication(s): by one month postoperatively, the patient reported substantial global improvement. Her headaches had resolved, breathing normalized, and balance had notably improved. At one year postoperatively, the patient continued to do well and imaging showed no signs of complications with all instrumentation in the correct positioning.

Case 11 [12]

Background: the authors of this paper published an article in 2021 detailing the case of a 38-year-old female with HCS who presented with a long-standing history of cervical and upper thoracic back pain that had more recently become increasingly progressive. She also noted worsening thoracic scoliosis. No signs of myelopathy were present. Imaging demonstrated severe thoracic scoliosis of 60 degrees, thoracic hypokyphosis, cervical kyphosis with swan-neck deformity, and CT findings concerning for significant osteoporosis. Flexion-extension radiographs demonstrated a fixed deformity. Due to her symptoms and the rate of progression from prior imaging, a staged anterior cervical reconstruction with posterior spinal cervicothoracic fusion was recommended.

Operation: stage 1a - posterior facetectomies at C4/5, C5/6, and C6/7. Stage 1b - anterior C5 and C6 corpectomy with expandable cage placement and C4-C7 anterior fusion. Stage 1c - C4-C7 posterior instrumentation. This multi-part surgery on the first day purposely placed her in greater malalignment to allow for global correction in the second stage of surgery.

Stage 2 - C4-T11 deformity correction with bilateral facetectomies from T5-T8 and C4-T11 posterior instrumentation. While most levels were instrumented bilaterally with pedicle screws, there were a few levels where her anatomy made this difficult, and hence one pedicle screw was used. Arthrodesis with the placement of local autograft, nonstructural allograft, and BMP followed. She was placed in a cervical collar for six months postoperatively.

Outcomes/complication(s): at one year postoperatively, the patient reported doing well with the resolution of cervical and thoracic back pain. Plain films showed excellent alignment and all instrumentation was in correct positioning.

Discussion

Our literature review yielded 11 case reports detailing patients with HCS and cranial or spinal pathology requiring an operation. Of these cases, six reported complications (54.5%) with four patients (36.5%) going on to require reoperation. The average follow-up of all reported cases with clear time frames was 14 months. Of the five cases with no reported complications, none had a follow-up over one year. Thus, the true complication rate is likely even higher. High complication rates in these patients are not surprising given the significant osteoligamentous impact and multisystem involvement in HCS. With only a few reported cases, a complete analysis is not possible, but several trends started to emerge in the discussion of the presenting concerns, operative considerations, and complications noted.

Presentation

Patients with HCS seem to present with cranial and spinal concerns with a few similar patterns. The bone softening seems to predispose patients to basilar invagination and brainstem compression, which is impressively severe in some cases. These patients may be at risk of syringomyelia, and clinical manifestations may include myelopathy in addition to lower cranial nerve dysfunction. Patients may also present with direct spinal pathologies. These appear to present either following trauma with patients having multiple significant fractures or as cervicothoracic deformity with expected clinical findings.

These presentations suggest that patients' cranial and spinal concerns are primarily driven by the severe osteoporosis seen in HCS. Though the exact mechanism of HCS in humans is unknown, mouse models with the NOTCH2 gain of function defects, which are seen in HCS, have been shown to result in osteoclastogenesis and induction of RANK-L production from osteoblasts [13,14]. This mechanism is congruent with the findings of fusion failure and osteolysis of fusion masses.

Pharmacologic intervention such as an augmentation method to fusion surgery with osteoclast-inhibiting, RANK-L inhibiting, or parathyroid hormone (PTH) analogs has not been studied in HCS patients, though animal models suggest the PTH analog teriparatide may increase postoperative fusion rates, whereas bisphosphonates decrease it [15-18]. In the few available case reports, only Vissiaranov et al. have reported the use of pharmacologic augmentation to fusion surgery. Their patient began IV bisphosphonate infusions every three months for two years following surgery before transitioning to infusions every six months. They reported no issues with fusion failure or osteolysis of fusion mass but described distal junctional kyphosis at four years postoperatively [11].

Fusion Failure/Osteolysis of Fusion Mass

Of the reported cases, the most common complication was the failure of fusion, occurring in two cases (18.1%). Fusion augmentation with osteoinductive agents such as DBM or BMP should be considered in these patients given their higher risk of fusion failure; however, it should be noted that no current evidence of their effects on this patient population is available. Hence, careful consideration of bone grafting materials should be made (Table 2).

**Table 2:** Bone grafting materials used with outcomes BMP: bone morphogenic protein; DBM: demineralized bone matrix

Author, case/operation/stage number	Bone grafting materials	Fusion achieved/sustained (yes/no)
Herscovici et al., case 1 [4]	Iliac autograft	No
Herscovici et al., case 2 [4]	Iliac autograft and allograft	No
Tanimoto et al., case 1 [5]	Iliac autograft	Yes
Murtagh-Schaffer and Moquin, case 1 [8]	Allograft, autograft, and DBM	No
Murtagh-Schaffer and Moquin, case 2 [8]	Allograft, autograft, and BMP	Yes
Mattei et al., case1, operation 1 [9]	Allograft	Yes
Woon and Mardjetko, case 1, stage 1 [10]	Allograft, DBM, BMP	Yes
Vissarionov et al., case 1 [11]	Autograft	Yes
Vissarionov et al., case 2 [11]	Autograft	Yes
Falls et al., case 2, stage 1 [12]	Local autograft, nonstructural allograft, and BMP	Yes
Falls et al., case 2, stage 2 [12]	Local autograft, nonstructural allograft, and BMP	Yes

In particular, BMPs have shown great promise. Augmentation with BMPs has been shown to significantly increase bone mass in the fusion bed and decrease the time to bony bridging after spine fusions in osteoporotic rats and hence has been proposed to improve the outcome of spinal fusions in osteoporotic patients [19]. While there is no direct evidence regarding BMP use in HCS patients, it is worth noting that in the three cases in which fusion failure or osteolysis of fusion mass occurred, BMP augmentation was not carried out. Murtagh-Schaffer and Moquin raise this same concern in their case report where their patient failed fusion after the first surgery, in which demineralized bone protein, as well as allograft and autograft bone, was used. On reoperation, they used BMP with their allograft and autograft bone and achieved successful fusion [8].

Cranial Suture Diastasis

As first shown by Woon and Mardjetko, a careful assessment of cranial sutures is important before performing any craniocervical fusion. Translation of forces in a cephalad manner may cause suture diastasis with resultant compression-related neurological symptoms. This was the result of their occipitocervical fusion [10]. Approximately two months after the surgery, shortly after the halo vest was removed and the patient began transitioning to a cervicothoracic orthotic, compression-related neurologic symptoms became apparent. This complication was addressed by the extension of the fusion construct to include the parietal bones as well as the frontal bones with titanium rods and mesh, which resolved the issue.

In our previous study, we were faced with a similar predicament as preoperative imaging showed unfused cranial sutures. During craniocervical fusion, we included the occipital bone as well as parietal and frontal bones in our construct, similar to Woon and Madjetko. Our patient continues to do well over one-year postoperatively.

Junctional Kyphosis/Failure

Instrumentation of the compromised bone in HCS patients raises concerns regarding transition zone failures. Osteopenia and osteoporosis have been found to be risk factors for proximal junctional kyphosis [4,5]. Of the 11 current case reports, junctional kyphosis is reported by Vissarionov et al., in which they describe a patient who experienced an abrupt decrease in the height of the anterior column immediately below the fusion construct four years after surgery. This patient was on regular bisphosphonate infusions. Deformity correction with the extension of the posterior fusion construct was successful with follow-up at eight months.

Anatomic Variations

Anatomic variations in HCS patients can present challenges for operative and postoperative management. Tanimoto et al. and Faure et al. have both reported difficulties in making a dural incision during suboccipital craniectomies due to large venous sinuses and relatively short occipital squama [5,6]. Tanimoto et al. have also reported that thin frontal bones prevented them from utilizing the halo vest postoperatively; they proceeded with the Philadelphia collar [5]. In the anterior cervical approach described by Mattei et al., substantial carotid dolichoectasia and significant bleeding from the porous and soft osseous structures complicated the operation [9]. In our previous paper describing a patient undergoing C4-T11 posterior fusion, we noted that the spinal anatomy made it difficult to place bilateral pedicle screws, and hence some levels only received unilateral screw placement [12].

Orthosis

Given the significant fusion-related concerns, many patients are managed with a postoperative orthosis. Halo placement has significant risks with fewer certain benefits in patients with unfused cranial sutures. Patients with significant deformities and atypical body habitus may also encounter issues with both cervical collars and thoracic orthoses. Where appropriate, we have found custom-molded orthosis to be particularly helpful in these patients.

Limitations

Throughout the drafting of this article, many limitations became apparent. Firstly, due to the rarity of the syndrome, there are very few cases available, limiting the ability to draw definitive conclusions. The short length of follow-up was also a limitation since the ability of these patients' osteoporotic bone to tolerate/accommodate instrumentation was inadequately assessed in this time frame. Lack of operative and case details was another limitation. Due to the minimal total cases available, it was important for us to be all-inclusive. This led to cases with a severe lack of details being included. Lastly, the lack of orthosis details was a weakness. Though most have authors included the type of orthosis used, details otherwise were sparse to nonexistent, and hence were unable to comment extensively on this aspect of care.

## Conclusions

HCS is a rare disorder that often affects the cranium and spine. Postoperative complication rates in these patients are high due to their underlying pathology. Though we were able to identify various trends in the surgical management of these patients, with only 11 cases available in the medical literature, much more remains to be learned to optimize outcomes.
